# Development of a Large SNP Genotyping Array and Generation of High-Density Genetic Maps in Tomato

**DOI:** 10.1371/journal.pone.0040563

**Published:** 2012-07-10

**Authors:** Sung-Chur Sim, Gregor Durstewitz, Jörg Plieske, Ralf Wieseke, Martin W. Ganal, Allen Van Deynze, John P. Hamilton, C. Robin Buell, Mathilde Causse, Saranga Wijeratne, David M. Francis

**Affiliations:** 1 Department of Horticulture and Crop Science, Ohio Agricultural Research and Development Center, The Ohio State University, Wooster, Ohio, United States of America; 2 TraitGenetics GmbH, Gatersleben, Germany; 3 Seed Biotechnology Center, University of California Davis, Davis, California, United States of America; 4 Department of Plant Biology, Michigan State University, East Lansing, Michigan, United States of America; 5 Institut National de la Recherche Agronomique, INRA, Unité de Génétique et d’Amélioration des Fruits et Légumes, Montfavet, France; 6 Molecular Cellular and Imagining Center, Ohio Agricultural Research and Development Center, The Ohio State University, Wooster, Ohio, United States of America; Nanjing Forestry University, China

## Abstract

The concurrent development of high-throughput genotyping platforms and next generation sequencing (NGS) has increased the number and density of genetic markers, the efficiency of constructing detailed linkage maps, and our ability to overlay recombination and physical maps of the genome. We developed an array for tomato with 8,784 Single Nucleotide Polymorphisms (SNPs) mainly discovered based on NGS-derived transcriptome sequences. Of the SNPs, 7,720 (88%) passed manufacturing quality control and could be scored in tomato germplasm. The array was used to generate high-density linkage maps for three interspecific F_2_ populations: EXPEN 2000 (*Solanum lycopersicum* LA0925 x *S. pennellii* LA0716, 79 individuals), EXPEN 2012 (*S. lycopersicum* Moneymaker x *S. pennellii* LA0716, 160 individuals), and EXPIM 2012 (*S. lycopersicum* Moneymaker x *S. pimpinellifolium* LA0121, 183 individuals). The EXPEN 2000-SNP and EXPEN 2012 maps consisted of 3,503 and 3,687 markers representing 1,076 and 1,229 unique map positions (genetic bins), respectively. The EXPEN 2000-SNP map had an average marker bin interval of 1.6 cM, while the EXPEN 2012 map had an average bin interval of 0.9 cM. The EXPIM 2012 map was constructed with 4,491 markers (1,358 bins) and an average bin interval of 0.8 cM. All three linkage maps revealed an uneven distribution of markers across the genome. The dense EXPEN 2012 and EXPIM 2012 maps showed high levels of colinearity across all 12 chromosomes, and also revealed evidence of small inversions between LA0716 and LA0121. Physical positions of 7,666 SNPs were identified relative to the tomato genome sequence. The genetic and physical positions were mostly consistent. Exceptions were observed for chromosomes 3, 10 and 12. Comparing genetic positions relative to physical positions revealed that genomic regions with high recombination rates were consistent with the known distribution of euchromatin across the 12 chromosomes, while very low recombination rates were observed in the heterochromatic regions.

## Introduction

Tomato (*Solanum lycopersicum* L.) has been a model species for basic studies in plant biology. The strength of genetic resources anchored to high-density maps has permitted the map-based cloning of genes involved in disease resistance [1]–[4], plant and fruit development [5], [6], and regulation of biochemical processes [7]. The first high-density genetic map for tomato consisted of over 1,000 restriction fragment length polymorphism (RFLP) markers segregating in an interspecific F_2_ population derived from a wide cross between *S. lycopersicum* and *S. pennellii* [8]. More recently, mapping studies have focused on polymerase chain reaction (PCR)-based markers with genetic maps of cultivated tomato developed using 344 Simple Sequence Repeat (SSR) and 793 Singe Nucleotide Polymorphism (SNP) markers [9] and integrated *S. lycopersicum* x *S. pimpinellifolium* maps based on 434 PCR-based markers [10].

The genomic resources available for tomato are rapidly expanding due to the increased throughput of next generation sequencing (NGS) technologies that have significantly reduced the cost and time of sequencing relative to the Sanger method and facilitated whole-genome sequencing, transcriptome profiling, and discovery of variation across genomes [11]–[13]. NGS has permitted genome-wide SNP discovery in many crop species including rice [14], [15], maize [16], durum wheat [17], sugarcane [18], soybean [19], [20], and potato [21]. In tomato, NGS of the transcriptome produced 17 Gb of sequence for six accessions and led to the identification of 62,576 non-redundant SNPs [22].

High-throughput SNP discovery has been paralleled by the development of genotyping platforms that permit cost-effective scoring of many thousands of SNPs in a highly parallel fashion [23], [24] facilitating high-density genetic map construction. For maize, an array consisting of 49,585 SNPs was used to develop two linkage maps with 20,912 and 14,524 markers, respectively [25]. In the age of incomplete genome sequences and partial physical maps, high resolution genetic maps remain an essential resource. Such maps help to improve genome assemblies, provide estimates of recombination relative to physical distance, and remain an essential feature for the dissection of complex traits. The information provides an essential guide to genomic assisted crop improvement, where recombination remains a constraint.

In order to facilitate genetic analysis and breeding, we developed the first large scale SNP genotyping array for tomato using 8,784 SNPs mainly discovered based on NGS-derived transcriptome sequences for six accessions [22]. Three high-resolution linkage maps were constructed using interspecific F_2_ populations to provide details of genetic order, recombination, and their position relative to the draft assembly of the tomato reference genome sequence. The SNP array and high-density linkage maps will be useful for population level analysis, trait discovery, and selection for cultivar improvement in tomato.

## Results

### SNP Array

We developed a genotyping array on the Illumina Infinium platform (Illumina Inc., San Diego, CA, USA) based on 8,784 SNPs. These SNPs represented a highly filtered and selected set, optimized for polymorphism detection among cultivated germplasm and spread throughout the genome. Of these, 7,720 SNPs (88%) passed manufacturing quality control (Table S1). A failure rate of 12% was considered normal and acceptable (less than 15% is expected according to the manufacturer). The scorable SNPs included 501 from candidate genes and 1,307 that were cross-validated with community data sets from TraitGenetics (Gatersleben, Germany), the French National Institute for Agricultural Research (Institut National de la Recherche Agronomique, INRA), and previously published SNPs [22], [26], [27] (Table S1).

### Genetic Map Construction

The widely used tomato reference population EXPEN 2000 was used to develop a SNP map (EXPEN 2000-SNP) based on 79 F_2_ individuals from a cross between LA0925 (*S. lycopersicum*) and LA0716 (*S. pennellii*). Among 7,720 scorable SNPs on the array, 3,640 were polymorphic between the parental lines and were analyzed in the mapping population. 3,503 SNP markers could be placed as codominant markers on the linkage map representing 1,076 unique map positions (genetic bins) with an average marker bin interval of 1.6 cM and the largest gap of 9.7 cM on chromosome 12 (Table 1 and Table S2). Each chromosome was covered by 70–125 genetic bins. We observed an uneven distribution of the markers on the array across all 12 chromosomes that was not in agreement with the reported chromosomal size [28]. For example, 252 SNPs covered 201.8 cM (113 genetic bins) on chromosome 1, while 466 SNPs covered 114.4 cM (82 genetic bins) on the cytologically smaller chromosome 11. For a confirmation of the chromosomal marker assignment, the SNP markers that were polymorphic between M82 and *S. pennellii* LA0716 were also localized on the reference introgression lines (ILs) [29] that were available for most of the tomato genome (except parts of chromosomes 4, 5, 8 and 9). The linkage mapping and the IL assignment were consistent with very few mismatches (Table S2).

**Table 1:** Number of SNP markers and coverage in cM of each chromosome in three linkage maps.

	EXPEN 2000 (LA0925 x LA0716)					EXPEN 2012 (Moneymaker x LA0716)					EXPIM 2012 (Moneymaker x LA0121)				
	No.	Unique	Coverage	Maker Interval (cM)		No.	Unique	Coverage	Maker Interval (cM)		No.	Unique	Coverage	Maker Interval (cM)	
Chr	Marker	Bin1	(cM)	Maximum	Average2	Marker	Bin	(cM)	Maximum	Average	Marker	Bin	(cM)	Maximum	Average
1	252	113	201.8	8.5	1.8	266	110	117.2	8.9	1.1	332	158	127.5	6.0	0.8
2	416	125	165.5	6.2	1.3	434	145	110.2	3.5	0.8	507	123	80.2	5.7	0.7
3	286	81	121.7	5.6	1.5	299	97	105.4	7.0	1.1	339	139	108.2	5.7	0.8
4	385	113	159.5	5.0	1.4	427	123	108.1	6.5	0.9	574	135	93.0	3.6	0.7
5	363	99	154.3	6.1	1.6	381	118	95.5	5.2	0.8	494	129	88.9	4.2	0.7
6	374	78	111.3	5.6	1.4	384	89	87.7	7.2	1.0	306	94	66.8	4.3	0.7
7	224	70	108.2	7.8	1.5	237	71	74.8	4.2	1.1	290	111	83.2	3.6	0.7
8	189	75	124.4	8.1	1.7	198	87	76.9	3.8	0.9	258	95	77.4	4.2	0.8
9	218	84	144.2	8.7	1.7	234	100	96.7	4.5	1.0	228	83	72.4	5.1	0.9
10	167	80	122.8	5.5	1.5	178	79	84.5	6.8	1.1	270	87	75.5	3.1	0.9
11	466	82	114.4	9.0	1.4	484	126	98.8	4.8	0.8	691	115	92.1	8.7	0.8
12	163	76	141.9	9.7	1.9	165	84	99.1	8.9	1.2	202	89	84.1	8.3	0.9
Total	3,503	1,076	1,669.9		1.6	3,687	1,229	1,154.6		0.9	4,491	1,358	1,049.2		0.8
			(1,252.4)3					(1,201.2)					(1,081.2)		

1 Unique map positions covered by SNP markers.

2 Average marker interval (cM) = coverage/number of unique bins.

3 Map length recalculated based on subsets of markers that were separated by at least 5 cM interval.

Since the EXPEN 2000-SNP map was based on relatively few (n = 79) individuals and the introgression lines did not cover all chromosomal regions, another linkage map (EXPEN 2012) was generated based on 160 F_2_ individuals derived from a cross between Moneymaker (*S. lycopersicum*) and LA0716. Of 3,770 polymorphic SNPs between the parental lines, 3,687 markers (1,229 genetic bins) were mapped with an average marker bin interval of 0.9 cM (Table 1 and Table S3). The two largest gaps of 8.9 cM each were on chromosomes 1 and 12. As in the EXPEN 2000-SNP map, the number of polymorphic markers for each chromosome did not correlate fully with the chromosomal size with the discrepancy being most pronounced for chromosome 1 and 11 (Table 1). Otherwise, the marker distribution between the two EXPEN maps was comparable.

In addition to the two EXPEN maps which were based on crosses between red-fruited species *S. lycopersicum* and the green-fruited *S. pennellii*, the EXPIM 2012 map was analyzed with 183 F_2_ individuals derived from a more narrow cross between Moneymaker (*S. lycopersicum*) and the red fruited LA0121 (*S. pimpinellifolium*). Among 4,792 polymorphic SNPs between the parental lines, 4,491 markers were mapped as codominant loci representing 1,358 genetic bins with an average marker bin interval of 0.8 cM and the largest gap of 8.7 cM on chromosome 11 (Table 1 and Table S4). The distribution of the SNP markers across all chromosomes was again similar to the other linkage maps (Table 1). The map of chromosome 1 consisted of 332 SNP markers covering 127.5 cM and 158 unique bins while the map of chromosome 11 consisted of 691 SNP markers covering 92.1 cM and 115 unique bins.

### Genetic Map Length

The total genetic distance of the EXPEN 2000-SNP map was estimated as 1,669.9 cM, or approximately 45% larger than the EXPEN 2012 map (1,154.6 cM) and 59% larger than the EXPIM 2012 map (1,049.2 cM) (Table 1). Although our estimate of genetic length for the EXPEN 2000-SNP map was marginally larger than expected based on previous estimates of genetic map length for this population (1,503 cM) [9], we were concerned about discrepancies in size between the three populations. One possible explanation for the observed increase in the amount of recombination in the EXPEN 2000-SNP map could be selection at gametophytic and post-zygotic stages, leading to distorted segregation and inflated estimates of recombination in that specific population. To address this possibility, we investigated whether there was an excess of chromosomes with distorted makers. Chromosomes 1, 10 and 11 contained a high proportion of distorted markers. A test for correlations between map expansion and distorted segregation did not support a positive relationship (m = −0.7; R^2^ = 0.19; *P* = 0.146) suggesting that distorted segregation was not responsible for the expanded map.

An alternative explanation for the map expansion observed for the EXPEN 2000-SNP map compared to the EXPEN2012 map relates to the large number of makers scored and the small population size. The accuracy of the calculations for genetic distance is influenced by population size since a falsely scored or incorrectly ordered marker has a larger effect in a smaller population. The EXPEN 2000-SNP map length may be overestimated as a result of population size which limits accurate estimation of marker order and genetic distances. To address this hypothesis, we repeated the EXPEN 2000-SNP map construction by selecting 307–325 markers that were separated by at least 5 cM interval and recalculated the genetic map. This resampling analysis led to estimates of map length that were reduced by an average of 25% (range 22–27%) relative to the EXPEN 2000-SNP map length based on all markers (Table 1). This reduction was not observed when the same approach was used in the EXPEN 2012 and EXPIM 2012 populations (Table 1). These results suggest that the small population size of the EXPEN 2000-SNP reference map limited the ability to accurately determine marker distance based on recombination when marker density was high.

The approach of creating a series of resampled maps allowed us to compare map length between the EXPEN 2012 and EXPIM 2012 populations. The 10% difference between the two maps was significant based on over 100 iterations. The EXPEN 2012 map was significantly (*P*<0.001) longer for chromosomes 2, 4, 5, 6, 9, 10, 11 and 12. The EXPIM 2012 map was significantly (*P*<0.001) longer for chromosome 1 and 7. No differences were detected for average distances on chromosome 3 and 8, though there may be differences in recombination length between the two maps for the arms of chromosome 8.

### Chromosome Assignment and Colinearity between Genetic Maps

**Figure 1:**
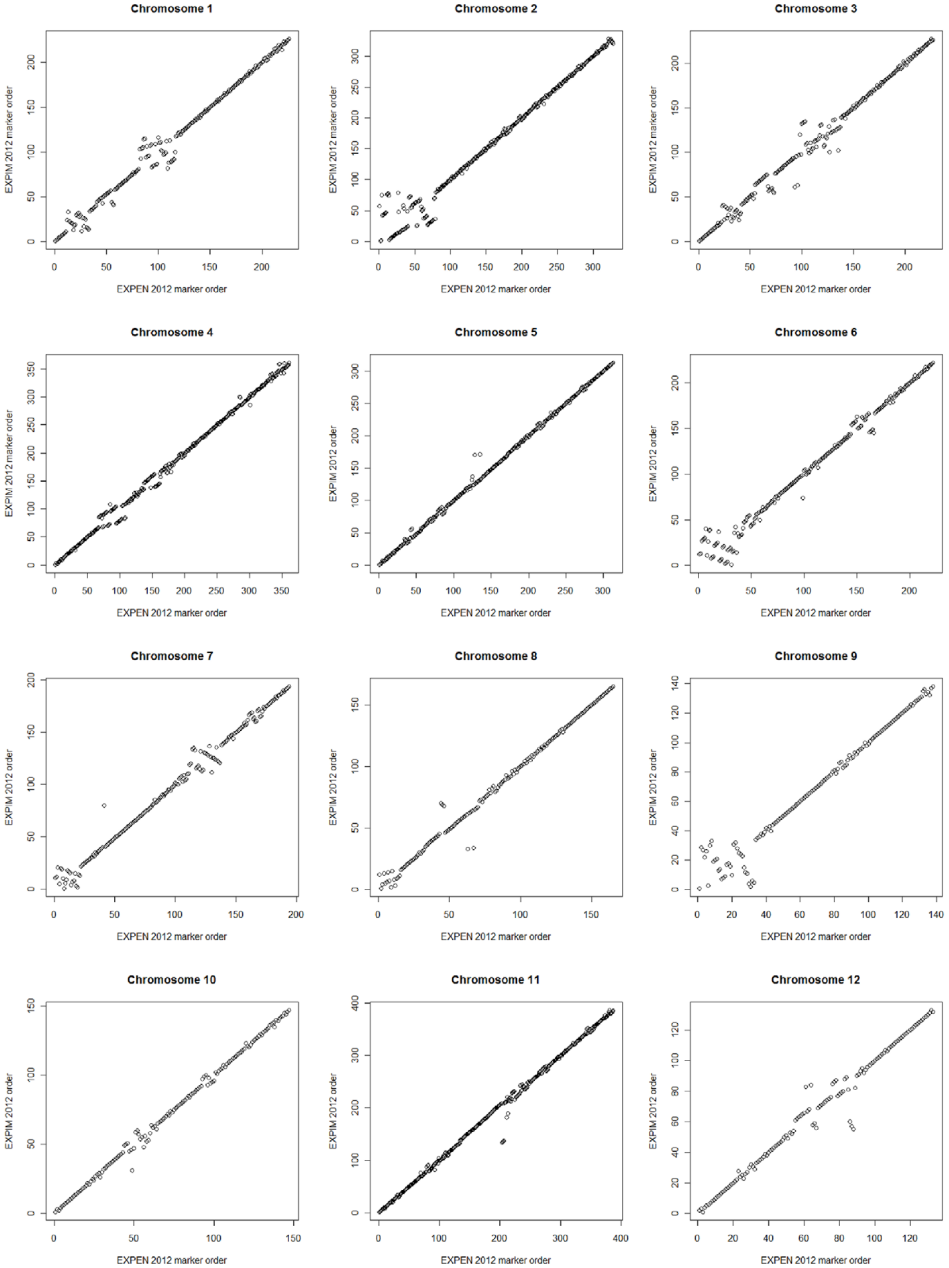
Regression of marker order between the EXPEN 2012 and EXPIM 2012 linkage maps. The 2,841 SNP markers common to both maps were ranked based on their map positions within chromosomes for each map and the rank orders were used for regression analysis.

The genetic positions of 5,621 SNP markers across 12 chromosomes could be determined with 3,149 markers in common between the EXPEN 2000-SNP and EXPEN 2012 maps; 2,509 markers in common between EXPEN 2000-SNP and EXPIM 2012 maps; and 2,841 markers in common between EXPEN 2012 and EXPIM 2012 maps (Table 2 and Tables S5, S6, S7). All of the shared markers showed highly conserved chromosome assignments. As with the individual maps, the number of markers in common for each chromosome varied and ranged from 106 on chromosome 12 to 413 on chromosome 11 (Table 2). In order to assess levels of colinearity between the linkage maps, the common markers were ranked based on their chromosome positions and their rank orders were used for regression analysis. High levels of colinearity (0.96–1.00 regression coefficients) were observed across 12 chromosomes between both EXPEN maps (Table 2). The EXPIM 2012 map showed coefficients of colinearity ranging between 0.85–0.99 for the EXPEN 2000-SNP comparison and 0.98–1.00 for the EXPEN 2012 comparison again indicating that the larger EXPEN 2012 map is most likely more accurate. Due to map quality, further comparative analysis was conducted only between the EXPEN 2012 and EXPIM 2012 maps which were of comparable population size (160 vs. 183 individuals). Plotting the common markers based on rank order revealed several regions with inverse marker orders, characterized by a strong linear correlation with a negative slope over short distances, between these linkage maps. Specifically, patterns on chromosome 1 (coordinates 20, 20), chromosome 3 (coordinates 40, 40), chromosome 6 (coordinates 20, 20), chromosome 7 (coordinates 5, 5 and 140, 140), and chromosome 9 (coordinates 20, 20) are consistent with inversions between the *S. pimpinellifolium* LA0121 and *S. pennellii* LA0716 parents (Figure 1). Regions on chromosome 1 (coordinates 100, 100) and chromosome 2 (coordinates 60, 60) highlight where marker order diverges, but evidence for a simple inversion based on a strong negative correlation is less robust (Figure 1).

**Table 2:** Colinearity between common markers for the three linkage maps.

	EXPEN 2000 vs. EXPEN 2012		EXPEN 2000 vs. EXPIM 2012		EXPEN 2012 vs. EXPIM 2012	
Chr	No. CommonMarker	Coefficient of Colinearity^1^	No. CommonMarker	Coefficient of Colinearity	No. CommonMarker	Coefficient of Colinearity
1	216	1.00	184	0.99	226	0.99
2	377	1.00	308	0.99	328	0.99
3	280	0.96	213	0.85	227	0.99
4	341	1.00	306	0.99	361	1.00
5	349	1.00	287	0.99	313	1.00
6	340	1.00	196	0.98	222	0.99
7	203	0.98	168	0.95	194	0.99
8	163	1.00	135	0.99	165	0.99
9	184	0.97	120	0.91	138	0.98
10	153	0.99	125	0.98	147	1.00
11	413	0.97	361	0.92	387	1.00
12	130	0.99	106	0.97	133	0.99
Total	3,149	0.99	2,509	0.96	2,841	1.00

1 Colinearity within each chromosome was assessed using common markers. The markers were ranked based on their map positions and the rank order was used for regression analysis, and expressed as R^2^.

### Comparison between Genetic and Physical Positions

In addition to the genetic map position, the physical positions of 7,666 SNPs were determined relative to the tomato reference genome sequence [30] (Table S1) and available through the Solanaceae Genome Network (SGN; http://solgenomics.net). A total of 758 Mbp of the tomato genome was covered by the SNP markers on the array with an average distance between markers of 0.12 Mbp (Table 3). Chromosome 1 showed the largest physical gap with no markers (7.36 Mbp) followed by a region on chromosome 12 (4.73 Mbp). The most markers (1,059 SNPs) were mapped on chromosome 11, which is cytologically one of the smallest tomato chromosomes [28].

**Table 3:** Physical coverage of 7,666 SNP markers.

			Marker Interval (Mbp)	
Chr	No. Marker	Coverage (Mbp)	Maximum	Average
1	554	90.13	7.36	0.17
2	871	49.48	3.83	0.06
3	679	64.70	4.38	0.10
4	861	64.01	2.03	0.08
5	783	64.91	2.70	0.09
6	748	45.88	2.66	0.06
7	443	64.98	3.93	0.15
8	396	62.97	2.95	0.16
9	473	67.60	4.52	0.15
10	405	64.74	3.17	0.16
11	1,059	53.28	2.37	0.05
12	394	65.32	4.73	0.17
Total	7,666	758.00		0.12

Flanking sequences of SNPs were used for the automatic batch BLAST against the Tomato WGS chromosome database (v SL2.40; http://solgenomics.net/organism/Solanum_lycopersicum/genome). The actual SNP positions relative to the Tomato genome sequence were identified using a custom Python script.

**Figure 2:**
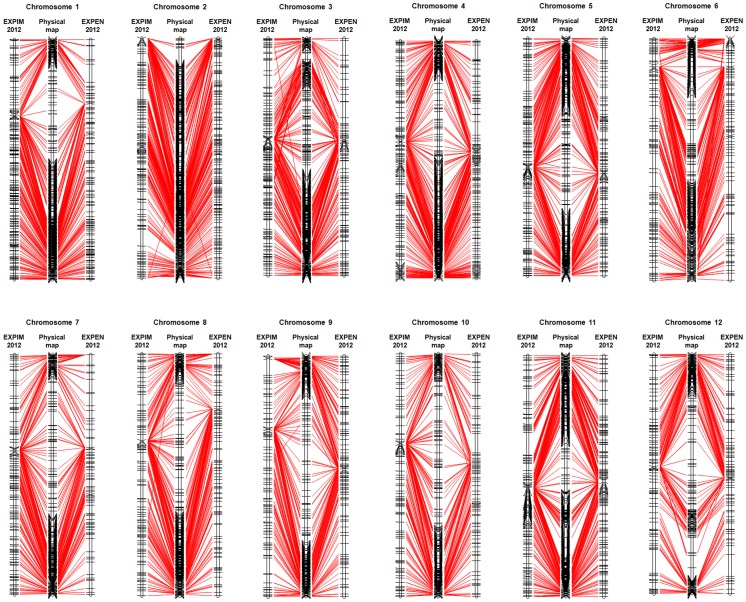
Comparative analysis of the EXPEN 2012 and EXPIM 2012 genetic maps relative to the draft assembly (v SL2.40; http://solgenomics.net/organism/Solanum_lycopersicum/genome **) of the tomato reference genome sequence.**

**Figure 3:**
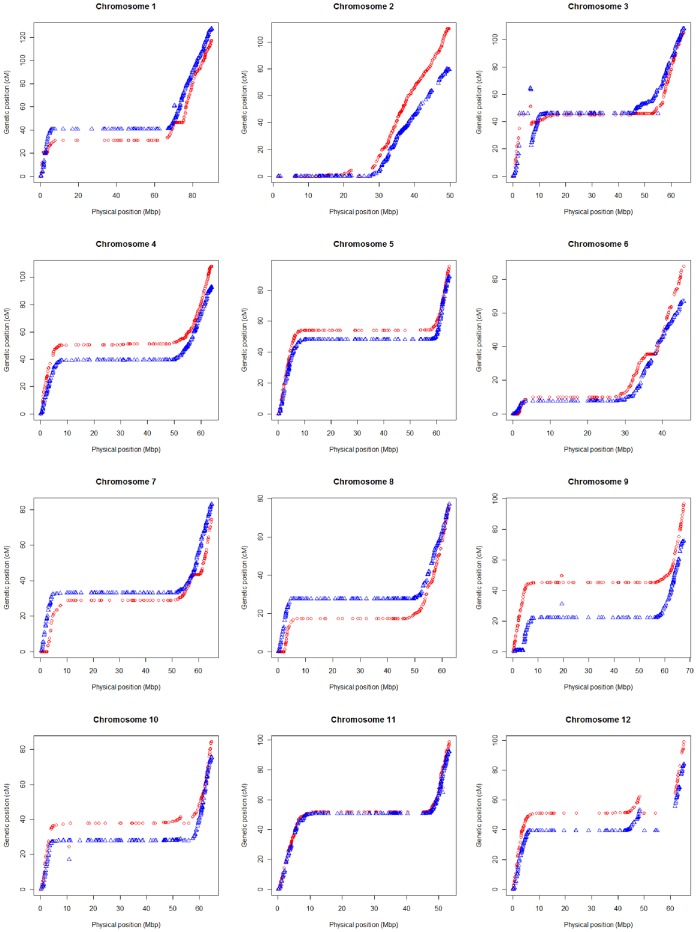
Relationship between genetic and physical positions within each chromosome. The genetic positions of SNP markers are indicated by red circles for the EXPEN 2012 population and blue triangles for the EXPIM 2012 population.

Among the 7,666 SNPs with physical positions, 5,296 SNP markers were mapped on one or both of the EXPEN 2012 and EXPIM 2012 genetic linkage maps (Table S8). These markers were used for comparative analysis of genetic and physical positions. We found that the vast majority (99.7%) of the SNPs in the linkage maps showed conserved chromosome assignments with the corresponding physical positions. Sixteen non-syntenic markers were not genetically mapped to the assigned physical chromosomes (Table S8). Among the 16 non-syntenic markers, there were eight markers mapped on both linkage maps with consistent chromosome assignments. For example, two markers were mapped genetically on chromosome 2, while they were physically placed on chromosomes 1 and 3. Further comparative analysis was conducted to determine colinearity within chromosomes. The two linkage maps revealed conserved marker order with the physical map for most regions of the genome, with chromosomes 4, 5, 8, and 11 having a very high level of colinearity (Figure 2 and Figure 3). A number of markers assigned to chromosomes 3, 10 and 12 in both linkage maps were not colinear with the physical map.

The meiotic recombination rate within each chromosome was estimated based on the 5,280 SNP markers with conserved chromosome assignments between genetic and physical maps. High recombination was found on the distal regions across all 12 chromosomes in both linkage maps, while recombination was suppressed in large regions that are most likely pericentromeric (Figure 3). The linkage maps also revealed similar patterns of variation in recombination rate between chromosomes. However, recombination rates appeared to be higher in the EXPEN 2012 map relative to the EXPIM 2012 map on chromosomes 2, 4, 5, 6, 9, 10, and 12, while the EXPIM 2012 population showed higher levels of recombination on chromosomes 1 and 7 (Figure 3). On chromosome 8, the overall rate of recombination appears similar, though the rate within each arm appears to differ between the two populations. These results are consistent with the results of the iterative mapping, described above. In addition, there was suppression of recombination specific to the EXPEN 2012 map on chromosome 1 (70–75 Mbp), chromosome 6 (36–38 Mbp), chromosome 7 (0–2 Mbp and 58–60 Mbp), and chromosome 8 (0–2 Mbp) (Figure 3). A recombination suppression specific to the EXPIM 2012 map was found on the region spanning 0–4 Mbp on chromosome 9.

## Discussion

The array with 7,720 scorable SNPs provides a valuable tool for high-throughput and cost-effective genotyping and mapping in tomato. The SNPs used for the array were derived from a computational pipeline based on cDNA sequences from six accessions including four representatives of large-fruited cultivated tomato, a cherry tomato and a closely related wild relative [22]. The array was optimized based on polymorphic SNP markers within cultivated lineages, allele frequency and genome coverage. In addition, 501 functional SNPs on the array were derived from candidate genes for traits such as disease resistance and carotenoid biosynthesis.

Given the physical length of chromosome 1 (largest chromosome), the number of markers is lower than expected while the number of markers on chromosome 11 is higher than expected. This distribution is not due to a lack of or excess of genes on these chromosomes but is likely due to the process of SNP marker selection. Alternatively, the distribution may reflect the introgression of highly polymorphic regions (e.g. containing disease resistance loci such as the *I2 Fusarium* resistance gene or the *Rx-4* and *Xv3* bacterial spot resistance genes on chromosome 11) that have created an ascertainment bias.

Despite the SNP selection for cultivated populations and the observed over- and under-representation, the SNP array provides a powerful resource for genetic map construction in interspecific populations. The EXPEN 2000 population has been used in the last ten years as a reference mapping population in tomato and 2,506 markers have been previously mapped (http://solgenomics.net) [31], [32]. With the SNP array, we mapped 3,503 SNP markers to this population. We also generated the EXPEN 2012 map for the *S. lycopersicum* Moneymaker x *S. pennellii* LA0716 population with 3,687 markers and the EXPIM 2012 map for the *S. lycopersicum* Moneymaker x *S. pimpinellifolium* LA0121 population with 4,491 markers. In total, we genetically positioned 5,621 SNP markers including common sets of 2,509–3,149 markers between the linkage maps.

The length of the genetic maps derived from the two EXPEN (*S. lycopersicum* x *S. pennellii*) populations differed from each other with the EXPEN 2000-SNP map length estimated to be 1,670 cM and the EXPEN 2012 map length as 1,155 cM. We investigated whether a possible reason for the differences in map length could be differential distortion due to gametic phase selection. If such distortion occurred in favor of LA0716 alleles on one portion of the chromosome and in favor of cultivated alleles on another, recombination would be overestimated in the progeny. Although the EXPEN 2000-SNP map showed a number of distorted markers on several chromosomes including chromosome 1 where genes affecting self and unilateral incompatibility are located [33], there was no correlation between segregation distortion and map expansion. Through the iterative analysis of marker subsets, we showed that the difference in genetic length between the two EXPEN maps was most likely due to the effect of scoring or ordering mistakes being amplified due to the small size the EXPEN 2000 population. Nevertheless both EXPEN maps are larger in terms of cM than the map from the EXPIM (*S. lycopersicum* x *S. pimpinellifolium*) population, and these differences were significant based on iterative estimates of map length. We expected that a genetic map generated from two more closely related parents would display a generally higher level of recombination. Our observation of greater map distance in the EXPEN populations is even more surprising given the likely existence of several small inversions between *S. lycopersicum* and *S. pennellii* which suppress recombination in these regions. Comparing the EXPEN 2012 and EXPIM 2012 maps suggests that there could be regions on chromosomes 1, 3, 6, 7, and 9 where small inversions differentiate LA0716 and LA0121. A paracentric inversion on the distal end of chromosome 7 was previously reported in *S. pennellii* LA0716 relative to *S. pimpinellifolium* LA1589 [34]. Further, cytogenetic analysis revealed that interspecific crosses between *S. lycopersicum* and *S. pennellii* can lead to changes in chromosome structure presumably due to inversions and translocations [35].

High-resolution genetic mapping with a large number of markers has helped to improve genome sequence assemblies in plants [25]. Comparison of genetic positions with physical positions provides an independent validation of reference genome sequence assembly. Most regions of the EXPEN 2012 and EXPIM 2012 linkage maps were fully colinear with the current assembly of the tomato reference sequence, suggesting a very good quality of the assembly. Sixteen markers with inconsistent chromosome assignment between genetic and physical maps were observed. Among them, eight markers had consistent chromosome assignments between the EXPEN 2012 and EXPIM 2012 maps, suggesting that the physical position may be incorrect or that the sequences are duplicated in the genome. Thus, the high-density genetic maps provide a guide to improve the assembly of genome sequence data. The genetic mapping of markers that are not present in the reference genome sequences can also improve the current genome assembly.

The comparisons between genetic and physical distances with several thousand markers reveal that there are similar patterns of variation in recombination rates along the tomato chromosomes. Strong recombination suppression occurs in the large pericentromeric regions within each chromosome. These regions represent repeat-rich and gene-poor heterochromatin encompassing 77% of the tomato genome [28], [36]. Such recombination suppression has been noted before for tomato and is also found in many other plant species [25], [37], [38] albeit often not as pronounced as in tomato.

With the availability of complete genome sequences, there is a tendency for genetic mapping to be relegated to a position of secondary importance. However, trait discovery, functional characterization, and crop improvement are largely dependent on recombination. Therefore, the construction of genetic maps which maximize the amount of recombination remains an essential tool in plant biology and plant breeding for precise and cost-efficient localization of traits and the generation of specific recombination events adjacent to interesting genes. Our data suggest that different crosses could reveal different general and location-specific levels of recombination, and that these differences are not necessarily related to the genetic distance between parents.

The SNP array and high-density genetic maps developed in this study will be useful in population level analysis of germplasm collections representing different market classes of cultivated tomato, regionally adapted populations and wild relatives. Other applications of the resource include genome-wide association mapping with high resolution and marker-assisted selection (MAS) for tomato breeding. For association mapping, accounting for population structure and/or familial relatedness is often necessary to avoid spurious marker-trait associations [39]. Large sets of genome-wide SNP markers will help to precisely estimate the relatedness and capture effects of quantitative trait loci (QTL). Association mapping has the potential to increase the efficiency of MAS by identifying markers tightly linked to traits of interest in germplasm panels that are directly relevant to plant breeders. In addition, the SNP array may facilitate genomic selection (GS) for plant breeding. As first suggested in animal improvement, GS seeks to predict the breeding value of individuals using markers distributed across the genome [40]. With the advent of high-throughput and cost-effective genotyping methods, GS is showing promise for improving complex traits in plant populations [41]–[43]. In summary, the SNP array provides a survey tool for the tomato research community and creates new opportunities for innovative strategies in both basic research and applied breeding.

## Materials and Methods

### Plant Material

For genetic mapping, we used 79 F_2_ progeny from the EXPEN 2000 population *S. lycopersicum* (LA0925) x *S. pennellii* (LA0716) which was previously published [31], [32]. To distinguish the new SNP map from the EXPEN 2000 reference map, we referred to the map described here as EXPEN 2000-SNP. The two other mapping populations were generated by TraitGenetics with the EXPEN 2012 consisting of 160 F_2_ progeny from a *S. lycopersicum* Moneymaker x *S. pennellii* (LA0716) cross and the EXPIM 2012 population of 183 F_2_ progeny derived from Moneymaker x *S. pimpinellifolium* (LA0121) [44]. The available *S. pennellii* introgression lines in the M82 background [29] were also used to compare marker assignment with the EXPEN SNP maps.

### SNP Array Development

SNPs for the array were selected based on a multi-tier strategy that was optimized for polymorphisms within and among cultivated types. Briefly, SNP discovery was based on the Genome Analyzer II-derived transcriptome sequences of four cultivated tomato accessions (NC84173, Fla.7600, OH08-6405, and OH9242), an *S. lycopersicum* var. *cerasiforme* accession (PI 114490), and an *S. pimpinellifolium* accession (PI 128216) [22]. SNPs were filtered such that any SNP within 50 bp of an intron/exon junction was removed and SNPs within 50 bp of a second polymorphism were excluded. The frequency of SNP occurrence among the six sequenced accessions was then assessed, with SNPs preferentially chosen based on their occurrence in multiple accessions. Genome coverage was assessed, and additional SNPs were selected to improve spacing across the genome. The research community provided a set of candidate genes of interest and 567 SNPs in the high confidence SNP set were located in these genes. Finally, SNPs were cross-validated with data sets from TraitGenetics, INRA, and previously published SNPs [26], [27]. We included 1,470 validated SNPs from these data sets on the array. A total of 8,784 SNPs detected with 10,000 probes were used to design the array (Table S1).

### Genotyping

Genomic DNA was isolated from fresh, young leaf tissue using a modified CTAB method [45]. Original DNA for the 75 F_2_ individuals of the EXPEN 2000 population was provided by Steven Tanksley (Cornell University, Ithaca, New York, USA) We also obtained DNA from the *S. pennellii* introgression lines in the M82 background from Dani Zamir (Hebrew University, Rehovot, Israel). Genotyping with the array was performed according to the manufacturer’s instructions for Illumina Infinium assay. The resulting intensity data was processed using the genotyping module v1.7.4 of the GenomeStudio software (Illumina Inc., San Diego, CA, USA) for SNP calling. In order to determine SNP genotype, a cluster file developed by TraitGenetics based on 92 hybrids facilitated allele calling in the Genome Studio software.

### Genetic and Physical Mapping

Three different software packages were used for mapping of the markers: JoinMap 4.0 [46], Map Manager QTXb20 [47], and MapChart 2.2 [48]. First, the genotyping data were transformed into the respective mapping data format (“ABH”, A = genotype parent 1, B = genotype parent 2, H = heterozygous). Subsequently, the JoinMap 4.0 program was used for verification of the segregation patterns, the formation of linkage groups and the preliminary positioning of the markers on chromosomes using the default grouping settings and the maximum likelihood mapping algorithm.

The final map position of the markers and the genetic distances between the markers were further optimized manually with respect to the number of crossovers (as low as possible) and the length of the linkage group (as short as possible) using the ABH mapping data file in Excel and MapManager QTX (settings: linkage evaluation F_2_ intercross, search linkage criterion *P* = 0.05, map function Kosambi, cross type line cross). The final map was drawn using MapChart 2.2.

In order to compare maps, an iterative approach was used in which at least 60 independent maps were created for each of the three populations. For each iteration, 217–325 markers were chosen based on a filter for 5 cM separation (determined by initial mapping). Map construction followed the steps described above, and comparisons between total map length and individual chromosome lengths were based on Analysis of Variance.

We determined the physical map position of the SNPs based on the flanking sequences used to develop the high-density Infinium array. These sequences were oriented relative to the genome sequence using the automated batch BLAST feature to search the Tomato WGS chromosome (v SL2.40; http://solgenomics.net/organism/Solanum_lycopersicum/genome) [30]. For a SNP with multiple BLAST hits, the best match was used to infer a map position. A custom Python script was then used to identify the actual SNP positions relative to the SL 2.40 genome sequence. We first calculated the 5′ flanking sequence length for each SNP. The script determined sequence orientation based on start and end positional information, and the SNP position was determined by adding or subtracting, depending on sequence orientation, the length of the flanking sequence to the corresponding subject start position. The accuracy of SNP positions was manually verified using a subset of data.

## Supporting Information

Table S1 **8,784 SNPs used for array development in this study.**
(XLSX)

Table S2 **3,503 SNP markers in the EXPEN 2000 (LA0925 x LA0716) linkage map and their assignment on the introgression line population of** ***S. pennellii*** **(IL).**
(XLSX)

Table S3 **3,687 SNP markers in the EXPEN 2012 (Moneymaker x LA0716) linkage map.**
(XLSX)

Table S4 **4,491 SNP markers in the EXPIM 2012 (Moneymaker x LA0121) linkage map.**
(XLSX)

Table S5 **3,149 SNP markers mapped on both the EXPEN 2000 and EXPEN 2012 linkage maps.**
(XLSX)

Table S6 **2,509 SNP markers mapped on both the EXPEN 2000 and EXPIM 2012 linkage maps.**
(XLSX)

Table S7 **2,841 SNP markers mapped on both the EXPEN 2012 and EXPIM 2012 linkage maps.**
(XLSX)

Table S8 **5,295 SNP markers with both genetic and physical positions.**
(XLSX)
